# A protocol for evaluating progressive levels of simulation fidelity in the development of technical skills, integrated performance and woman centred clinical assessment skills in undergraduate midwifery students

**DOI:** 10.1186/1472-6920-13-72

**Published:** 2013-05-24

**Authors:** Susannah Brady, Fiona Bogossian, Kristen Gibbons, Andrew Wells, Pauline Lyon, Donna Bonney, Melanie Barlow, Anne Jackson

**Affiliations:** 1 School of Nursing & Midwifery, The University of Queensland, Ipswich Campus, Salisbury Road, Ipswich, QLD 4035, Australia; 2 School of Nursing and Midwifery, The University of Queensland, Herston Campus, Edith Cavell Building, Fourth Avenue,, Brisbane, Herston, QLD 4029, Australia; 3 Mater Education, Mater Health Services, Mater Education Practice Improvement Center (MEPIC), Corporate Services Building, Level 4, Raymond Terrace, South Brisbane, QLD 4101, Australia; 4 Mater Research Office, Mater Medical Research Institute, South Brisbane, QLD 4101, Australia

**Keywords:** Clinical assessment, Education, Global rating scale, Integrated procedural performance instrument, Midwifery, Simulation, Simulation fidelity, Vaginal examination, Woman centred care

## Abstract

**Background:**

Simulation as a pedagogical approach has been used in health professional education to address the need to safely develop effective clinical skills prior to undertaking clinical practice. However, evidence for the use of simulation in midwifery is largely anecdotal, and research evaluating the effectiveness of different levels of simulation fidelity are lacking.

Woman centred care is a core premise of the midwifery profession and describes the behaviours of an individual midwife who demonstrates safe and effective care of the individual woman. Woman centred care occurs when the midwife modifies the care to ensure the needs of each individual woman are respected and addressed. However, a review of the literature demonstrates an absence of a valid and reliable tool to measure the development of woman centred care behaviours. This study aims to determine which level of fidelity in simulated learning experiences provides the most effective learning outcomes in the development of woman centred clinical assessment behaviors and skills in student midwives.

**Methods/Design:**

Three-arm, randomised, intervention trial.

In this research we plan to:

a) trial three levels of simulation fidelity - low, medium and progressive, on student midwives performing the procedure of vaginal examination;

b) measure clinical assessment skills using the Global Rating Scale (GRS) and Integrated Procedural Performance Instrument (IPPI); and

c) pilot the newly developed Woman Centred Care Scale (WCCS) to measure clinical behaviors related to Woman-Centredness.

**Discussion:**

This project aims to enhance knowledge in relation to the appropriate levels of fidelity in simulation that yield the best educational outcomes for the development of woman centred clinical assessment in student midwives. The outcomes of this project may contribute to improved woman centred clinical assessment for student midwives, and more broadly influence decision making regarding education resource allocation for maternity simulation.

## Background

### Woman centred care in midwifery

Midwifery is as much an art as it is a science [1]. Whilst the development of proficient psychomotor technical skills is critical for safe midwifery practice [2], equally important is the development of non-technical skills. These non-technical skills include proficiency in global clinical performance, communication, documentation and, essential to midwifery practice, an acknowledgement that pregnancy and birth is woman centred. Woman centred care is practiced in such a way that it values, supports and respects the woman, her ideas, questions, preferences, choices and decisions. Woman centred care is effected in an environment of shared power and responsibility between the woman and her midwife [3,4]. Woman centred care in clinical assessment is safe, supportive, and gentle. Woman centred care is an art that is handed down from one generation of midwives to another, the foundations of which are embedded in undergraduate midwifery courses, and strengthened through shared experiences in clinical practice [4].

### Simulation in the midwifery context

Simulation has been a key educational technique in medical and nursing training [5] in which elements of the real world are appropriately integrated to learning. Midwifery education has employed simulation methodology since the 1700’s when Madame de Coudray, a royal midwife, was commissioned by King Louis XV to travel and teach student midwives and used life size models of women she made from leather, bones and fabric [6]. In the following centuries, torso models and manikins were used to teach midwifery clinical skills such as abdominal palpation, mechanisms of labor and neonatal resuscitation.

### Simulation fidelity in the midwifery context

Simulation techniques vary in their levels of fidelity or realism and this is not dependent upon the level of technology. High fidelity may be achieved through low technology simulations and conversely the use of high technology simulators does not guarantee high fidelity. Simulation fidelity varies in midwifery education including the use of scenarios, peer-to-peer learning, partial task trainers, computerized task trainers, screen based simulations, virtual reality, haptic systems, standardized patients and full scale simulation and is used extensively in Australian midwifery programs to teach both technical and non-technical skills [7].

A study by McKenna et al. [8], looked at the perceptions of Australian midwifery educational leaders, who believed simulation could offer educational opportunities to support the development of competent midwives as long as the realism of the equipment was appropriate. This study recommended further investigation regarding the effect of differing levels of simulation fidelity on educational outcomes to ensure the most appropriate use of limited resources.

Recently simulation has been used to address the need to develop clinical skills prior to clinical practice to ensure patient safety, enhance clinical confidence prior to clinical exposure and reduce pressure on over-worked clinical environments [5,9]. Despite this change in educational methodology, minimal documentation regarding the effectiveness of these educational methods exists, including the effect on the development of woman centred care skills in student midwives.

In our study high fidelity will be achieved using simulated standardized patients, whom are individuals trained to act as a real patient to simulate a set of symptoms or problems [10]. Medium fidelity will be achieved using part-task trainers in combination with an innovative hybrid standardized patient in the format of a life size poster of a pregnant woman known affectionately as ‘Flat Maggie’. ‘Flat Maggie’ was developed internally by Pauline Lyon and Penelope Buntine, as a visual simulation of a pregnant woman in order to increase the realism of the learning experience. Low fidelity will be achieved by the use of part-task trainer only.

### Wom*a*n centred care vs. wom*e*n centred care

Within the literature a great deal is written about the primacy of woman centred care in the practice of midwifery [3,4,11-15]. The concept of woman centred care is central to midwifery practice and underpins the philosophy statement of the International Confederation of Midwives [13] and the Australian College of Midwives [14]. Debate exists around wom***e***n centred care and wom***a***n centred care with the former relating to institutional or organisational practices that cater for particular groups of women and the later to practices demonstrated by the individual midwife that cater for an individual woman [4]. Regardless, to promote the safe and effective care of women and families, the development of woman centred care behaviours are essential for student midwives to care for women in a holistic manner.

### Measuring midwifery skills

A variety of tools exist to measure development of task-based skills in health care professionals, including the Global Rating Scale (GRS) for the evaluation of technical skills [16] and the Integrated Procedural Performance Instrument (IPPI) [17]. A useful framework for assessing the development of practice behaviours in student midwives is the Australian Nursing and Midwifery Council National Competency Standards for the Midwife [18]. However, a review of the literature demonstrates the absence of a valid and reliable tool to measure the development of woman centred care behaviours. So piloting a tool to measure woman centred care behaviours is an important secondary aim in this research. These essential woman centred care behaviours are seen as integral in any midwifery care episode and therefore should be embedded, developed and assessed as part of the education of student midwives.

A proposed conceptual framework for woman centred care comprises eight key concepts which have been identified and defined [4,19,20]. These concepts have subsequently been linked to competencies, elements and cues from the National Competency Standards for the Midwife [18] and translation into practice behaviors identified and described. For example in the Woman centred care scale (WCCS) under the construct of Midwife /Mother relationship, the student performing the procedure will need to demonstrate skills in accurately interpreting the woman’s sensory cues during the procedure and demonstrating compassionate responses to performing care in order achieve a strongly agree standard on the tool. These practice behaviours can be directly linked the National Competency standards for the Midwife 2.1 and 3.3 [18]. These eight key identified and defined concepts of midwifery care form the basis of the tool to assess performance of woman centred care behaviours during practice of clinical technical skills. Further details of the development, content validation and refinement of this tool will be reported elsewhere.

### Aim(s) of the study

This research aims to determine the effect of progressive simulation fidelity on the development of global and integrated technical skills and woman centred clinical assessent skills in student midwives. The primary aim is to determine which level of fidelity of simulated learning experiences (low, medium or progressive fidelity) provides the most effective learning outcomes. The secondary aim of this study is to pilot a tool for assessing woman centred care behaviors in undergraduate midwifery students.

### Research question

Which level of fidelity of simulated learning experiences contributes to better global, integrated and woman centred care clinical assessment in student midwives?

### Hypotheses

#### Primary hypotheses

1. Students who undertake progressive fidelity experience will demonstrate higher Global Rating Scale (GRS) scores than students who undertake lower fidelity experience.

2. Students who undertake progressive fidelity experience will achieve higher Integrated Procedural Performance Instrument (IPPI) scores than students who undertake lower fidelity experience.

#### Secondary hypotheses

1. Students who undertake medium fidelity experience will demonstrate similar Woman Centred Care Scale (WCCS) scores as those that have progressive fidelity experience.

2. Students who undertake progressive fidelity experience will achieve higher Woman Centred Care Scale (WCCS) scores than students who undertake lower fidelity experience.

## Methods/Design

The three arm intervention trial replicates the methodology employed in a study evaluating the effectiveness of progressive fidelity in the acquisition of Intravenous cannulation technical and non-technical skills in medical students [21].

### Sample & intervention

In our study, first year Bachelor of Midwifery (BM) and Dual Degree (DD) Bachelor of Nursing and Bachelor of Midwifery students from the University of Queensland (UQ), who have yet to receive theoretical instruction on vaginal examination will be recruited. Following random allocation via a computer generated block randomization schedule, participants will be assigned to one of three intervention arms. The participants will receive identical theoretical instruction in the performance of vaginal examination. All participants will be provided with a list of process goals, including woman centred care behaviours developed from published guidelines and will be briefed on the nature and expectations of the simulation experience by a simulation expert.

Senior Bachelor of Midwifery (BM) and Dual Degree (DD) Bachelor of Nursing and Bachelor of Midwifery students (3rd year or 4th year students respectively) will be recruited to act in the role of the standardized patient. These students will be provided with training and scripts to be followed as standardized patients and will be briefed on the nature and expectations of the experience by the simulation expert. The standard scenario, including all associated history, details and expected responses to questions, will be developed by the group participating as standardized patients, guided by the simulation expert.

To achieve similar statistical significance demonstrated in the Brydges et al. [21] methodology it was determined that;

#### Intervention arm 1

**Figure 1:**
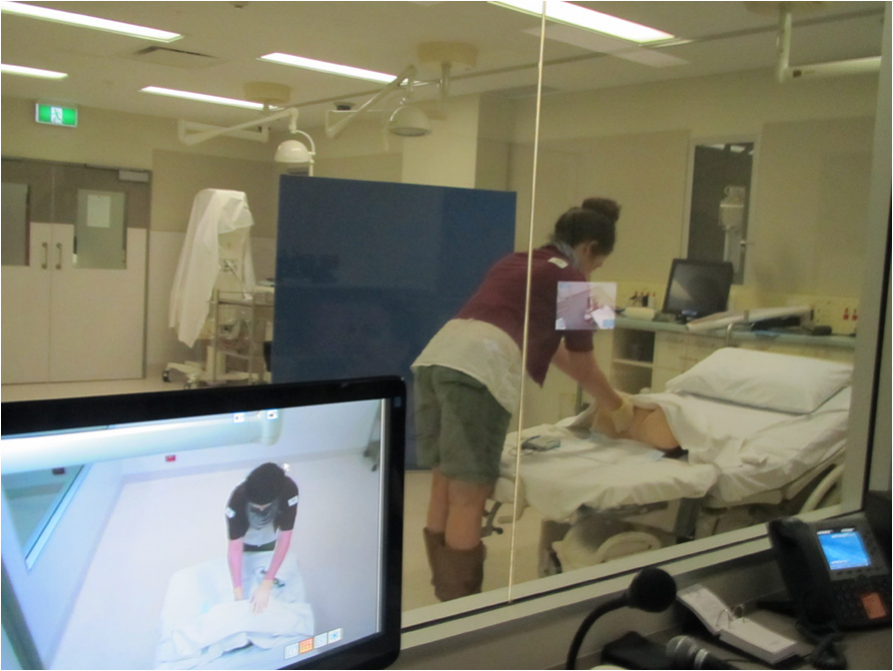
**Intervention arm 1.** Low fidelity will be achieved by the use of part-task trainer only, for the clinical procedure.

Intervention Arm 1 (n = 35) will receive **low fidelity** skills training using a **partial task trainer** positioned on a standard hospital birth-suite bed. Partial task trainers are designed to replicate a part of a system or process, in this research, Model-Med Charlie Obstetric Trainer® is used (Figure 1).

#### Intervention arm 2

**Figure 2:**
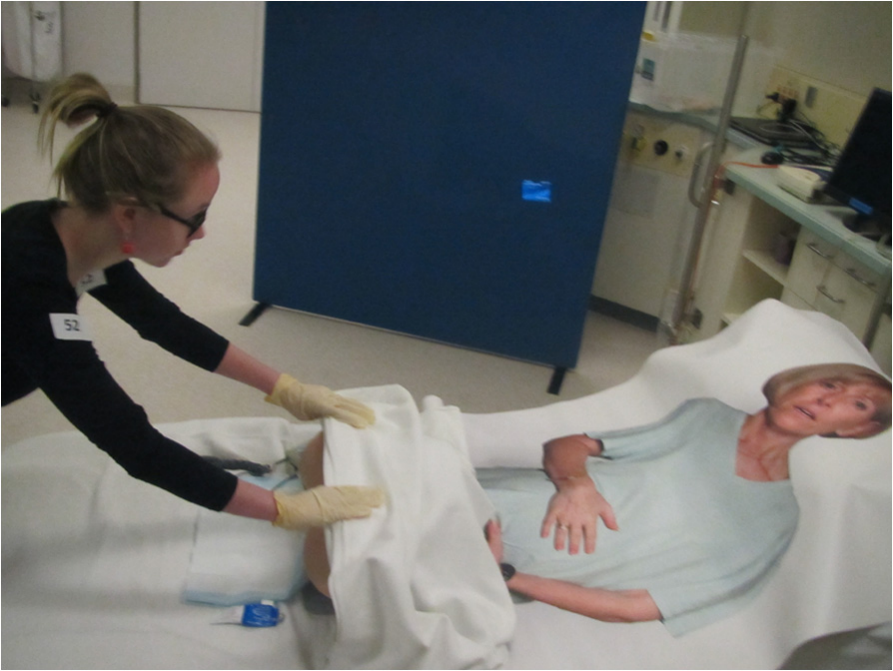
**Intervention arm 2.** Medium fidelity will be achieved using part-task trainers in combination with an innovative hybrid standardized patient in the format of a life size poster of a pregnant woman known affectionately as ‘Flat Maggie’.

Intervention Arm 2 (n = 35) will receive **medium fidelity** skills training using the same **partial task trainer** as provided in Intervention Arm 1, but with the addition of a ‘Flat Maggie’ positioned on a standard hospital birth-suite bed. ‘Flat Maggie’ will be draped over pillows in a bed to visually simulate a pregnant woman and the partial task trainer will be appropriately positioned in relation to the ‘Flat Maggie’ for the skill to be practiced (Figure 2).

#### Intervention arm 3

**Figure 3:**
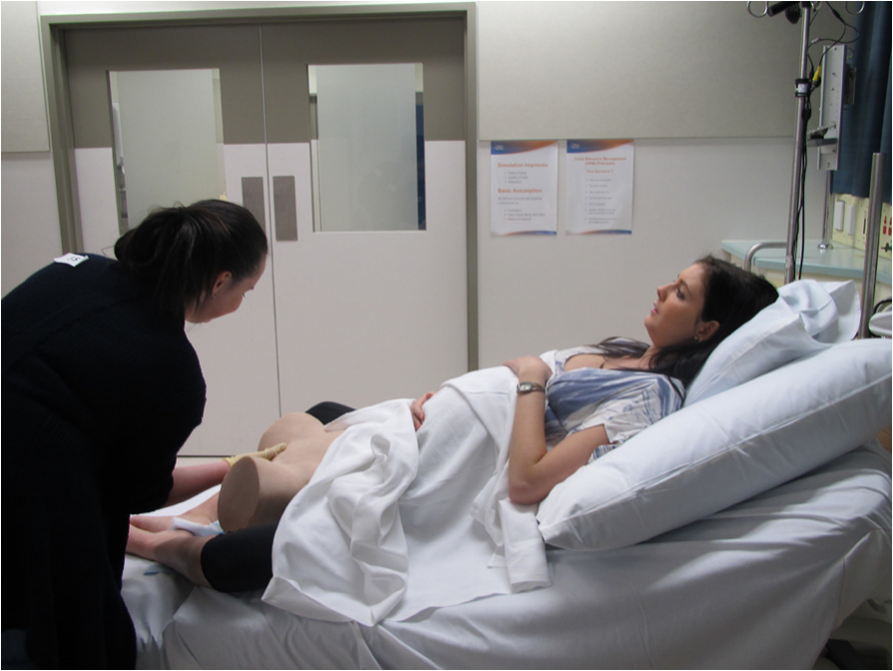
**Intervention arm 3.** High fidelity will be achieved using simulated standardized patients who are final year midwifery students trained to act as a real patient to simulate a set of symptoms or problems, with an appropriately positioned partial task trainer.

Intervention Arm 3 (n = 35) will receive **progressive fidelity** skills training through **low** – **partial task trainers**, **medium** – ‘**Flat Maggie’ with the partial-task trainer**, and then to **high fidelity – Standardized patients with the partial-task trainer** positioned on a standard hospital birth-suite bed. Standardized patients will be senior midwifery students who act the part of the pregnant woman, using a consistent and standard approach. It is important to note that standardized patients **will not** be subject to vaginal examination, rather they will act the role of the woman and a partial task trainer will be appropriately positioned for the skill to be performed (Figure 3).

### Sample size and statistical power

To ensure that the type I error rate for the entire study is 0.05, the sample size calculations used a type I error rate of 0.025 (Bonferroni’s correction) to account for the two primary outcome measure; GRS and IPPI scores.

#### GRS

Based on data from Brydges et al. [21], the expected sample standard deviation to be used in the sample size calculation was 3.6. Along with this parameter, a power of 80%, type I error of 0.025, and the assumption that the average GRS scores in the progressive, medium and low fidelity groups will be 11, 10 and 8, 102 participants are required. Inter-rater reliability (intraclass correlation = 0.76) and internal consistency (Cronbach’s alpha = 0.75) of the GRS have been demonstrated in published research studies [22].

#### IPPI

Based on data from Brydges et al [21], the expected sample standard deviation to be used in the sample size calculation was 18. Along with this parameter, a power of 80%, type I error of 0.025, and the assumption that the average IPPI scores in the progressive, medium and low fidelity groups will be 40, 33 and 25, 105 participants are required. Validation of the IPPI has been demonstrated in previous research studies; a recent study reported inter-rater reliability (intraclass correlation = 0.73), internal consistency (Cronbach’s alpha = 0.65) and discriminant validity (p < 0.001) [22].

#### WCCS

A conceptual framework has been proposed for woman centred care in which eight key concepts have been identified and defined, drawing on ANMC [18], Brison [19], Leap [4] and Leap [20]. The key concepts are the foundations of woman’s sphere, holism, self-determination, the sharing of power in the relationship, individuality, continuum, informed decision making and the midwife-woman relationship. These concepts are linked to competencies, elements and cues from the National Competency Standards for the Midwife [18] and translation to practice behaviors identified and described. This forms the basis of the tool to assess performance of these behaviours during practice of the clinical technical skill vaginal examination. As no comparable tool exists within the literature, the WCCS will be piloted in this research study.

Therefore, 105 participants are required in total (35 per group) for this study.

### Statistical methods

The descriptive characteristics of each of the three groups will be presented; continuous characteristics will first be assessed for distribution using histograms and the Shapiro-Wilk test. If the data is normally distributed, the mean and standard deviation will be presented, and for data that is not normally distributed, the median and interquartile range will be reported. Numbers and percentages will be used for categorical data. Comparison of the primary outcome measure amongst the three groups will be undertaken using either analysis of variance (ANOVA) or Kruskal-Wallis testing, dependent on the distribution of the outcome. If a statistically significant difference is found (at the 0.025 level), appropriate post-hoc testing will be conducted to determine which groups are different.

### Data collection

Participants’ performance of the skills will be video recorded and downloaded on to a computer and stored on a secure server. Videos will only be viewed by AW and two clinical Midwife Research Assistants external to the research team. Their scores for the three instruments will be entered directly into a database set up using Remark®.

The Global Rating Scale (GRS) [16] will be used to evaluate technical skills acquisition, the Integrated Procedural Performance Instrument (IPPI) [17] will be used to evaluate skills integration, and the Woman Centred Care Scale (WCCS) will be piloted. Inter-rater reliability and intra-rater reliability testing will be integrated into the design of this research.

Following completion of the activity and debriefing, all participants will evaluate the simulation experience using a University of Queensland procedural skills workshop evaluation.

Analysis of data will be guided by the specific instruments used. Qualitative data that emerges from debriefing or evaluation of the simulation experience will be thematically analyzed.

#### Study setting/location

The study will be conducted at the Mater Education Practice Improvement Centre (MEPIC), Raymond Terrace, South Brisbane, Queensland, Australia.

#### Study population

First year Bachelor of Midwifery (BM) and Dual Degree (DD) Bachelor of Nursing and Bachelor of Midwifery students from the University of Queensland (UQ), who have yet to receive theoretical instruction on vaginal examination will be recruited. Students will be provided with full details of the study and receive a participant information letter. Recruitment of participants will be undertaken by a University of Queensland (UQ) staff member who is not involved in the Bachelor of Midwifery or Dual Degree programs and is not a member of the investigation team. The students who agree to be involved in the study will undertake the planned simulation instruction and will be randomly assigned to one of the three intervention arms.

Senior Bachelor of Midwifery (BM) and Dual Degree (DD) Bachelor of Nursing and Bachelor of Midwifery students (3rd year or 4th year students respectively) will be recruited to act in the role of the standardized patient. They will be provided with full details of the study and receive a participant information letter. Recruitment will be undertaken by a University of Queensland (UQ) staff member who is not involved in the Bachelor of Midwifery or Dual Degree programs and is not a member of the investigation team. Those students who agree to be involved in the study will be given training to undertake the role of the standardized patient in the planned intervention by a simulation expert, and will receive a certificate of acknowledgement.

#### Participant inclusion criteria

In order to be included in the intervention component of the study participants will be volunteer first year Bachelor of Midwifery or Dual Degree students who have yet to receive instruction in performing vaginal examination. Participants will likely be between the ages of 17 - 60 years and either female or male.

In order to be included in the standardized patient group participants will be volunteer senior midwifery students in either the Bachelor of Midwifery or Dual degree (in 3rd or 4th year respectively); these students will receive both instruction in, and experiences of, performing vaginal examination and recognizing woman centred care behaviors.

#### Participant exclusion criteria

Students who are not enrolled in a midwifery program will be excluded from participating. Participation in this simulation project is voluntary, and participants will be free to withdraw at any time without consequence.

#### Safety considerations

The research design will minimize the risks of harm or discomfort to participants and ensure the selection, recruitment, exclusion and inclusion of participants is fair and will not overburden any group of students or staff. Participant information sheets will clearly outline the aim, benefits and involvement in the study, including adding value to future curriculum development processes at the University of Queensland. The research design will carefully address the issue of consent. Participants will be given sufficient information about the research in order for them to give informed consent and all participants are to be advised that they are free at any time to withdraw consent to further involvement in the research without consequence.

There is the unlikely potential for first year student participants to experience stress / distress / discomfort similar to that experienced in real life clinical practice. Third and fourth year student participants may also have the unlikely potential to experience stress / distress / discomfort similar to that experienced by individuals engaging in role play/simulation. No invasive procedures will be performed on human volunteers, using simulation models / manikins in these circumstances. Participants will all be midwifery students who are expected to engage in clinical practice experience in real life settings as part of their preparation for professional life on graduation.

Debriefing will be built into the simulation experience and will be conducted by a simulation expert to minimize the risk of distress. The simulation scenarios will also be observed by room monitors whom will be experienced clinicians able to intervene if any undue participant stress / distress or discomfort is identified. All participants will have information given to them that states if they experienced psychological stress / distress or discomfort, they may talk privately with any member of the research team to gain assistance, or access existing support services from either Mater Health Services or the University of Queensland freely and without prejudice.

### Ethical considerations

This research has secured ethical clearance from the Human Research and Ethics Committees of the University of Queensland (Protocol Ref No 2012000889) and the Mater Health Services, Brisbane (Protocol Ref No 2012-53).

## Discussion

The outcomes of this project will contribute to improved clinical assessment skills in student midwives particularly in relation to global and integrated performance, and more broadly influence decision making regarding education resource allocation for maternity simulation. The pilot of the WCCS tool will support its continued development and assist in the measurement of core concepts of midwifery care in undergraduate midwifery curricula.

This research will contribute an important evaluation of an innovative teaching and learning strategy in undergraduate professional curricula. The research will add further knowledge in the field of clinical education, with specific knowledge regarding the use of simulation fidelity to guide appropriate educational outcomes for undergraduate midwifery students.

## Abbreviations

ACM: Australian college of midwives; ANMC: Australian nursing and midwifery council; ATSI: Aboriginal and torres strait islanders; BM: Bachelor of midwifery; DD: Dual degree; GRS: Global rating scale; HWA: Health workforce Australia; ICM: International confederation of midwives; IPPI: Integrated procedural performance instrument; MEPIC: Mater education practice improvement center; MEL: Mater education limited; UQ: University of Queensland; VE: Vaginal examination; WCCS: Woman centred care scale

## Competing interests

The authors declare that they have no competing interests.

## Authors’ contributions

SB, FB, KG, AW, PL, DB, MB, AJ, contributed to the conception and design of this study; SB and FB devised study protocol AW drafted the manuscript; FB, SB, KG, DB, MB, AJ, PL, AW critically reviewed the manuscript; SB and FB conceptualized and developed the WCCS. All authors read and approved the final manuscript. PL is the simulation expert referred to through the research paper. The Principal Investigator, SB is leading this research as a part of a University of Queensland New staff start up grant.

## Authors’ information

Susannah Brady- is an advanced practice midwife and the Program Director for the Bachelor of Midwifery and Dual degree at The University of Queensland. Susannah was a member of the research team that conducted the Health Workforce Australia Midwifery Curriculum Simulation Project, and has a long standing interest in simulated learning.

Fiona Bogossian is the Director of Research at The University of Queensland. Fiona has 25 years experience in the tertiary education of nurses and midwives. In 2011 she led a team of researchers conducting the Health Workforce Australia Midwifery Curriculum Simulation Project.

Kristen Gibbons -is the Data Management and Analysis Team Leader, and consultant Biostatistician, for the Mater Research Office, Mater Medical Research Institute.

Andrew Wells has a long history of education in nursing and simulated learning. He currently works as a project manager for the Mater Health Services.

Pauline Lyon-is an advanced practice Midwife, Clinical Midwifery Consultant, Midwifery Educator and Simulation Education Coordinator at the Mater Education Practice Improvement Center.

Donna Bonney -is the Chief executive officer at the Mater Education Centre and the Director of Learning and Development in the Mater Health Services.

Melanie Barlow is an RN and Education Programs Manager and has experience as a Nurse Educator delivering simulation based education. Melanie has undertaken Instructor training in simulation – Nationally and Internationally (Institute of Medical Simulation, Harvard).

Anne Jackson is an advanced practice midwife with experience in education.

## Pre-publication history

The pre-publication history for this paper can be accessed here:

http://www.biomedcentral.com/1472-6920/13/72/prepub
